# Risk factors for mortality among inpatients with smear positive pulmonary tuberculosis

**DOI:** 10.12669/pjms.35.5.919

**Published:** 2019

**Authors:** Sidra Hameed, Faisal Faiyaz Zuberi, Sagheer Hussain, Syed Khalid Ali

**Affiliations:** 1 Dr. Sidra Hameed, MBBS, DTCD. Chest Unit-II, Ojha Institute of Chest Diseases Dow University of Health Sciences, Karachi, Pakistan; 2 Dr. Faisal Faiyaz Zuberi, MBBS, FCPS (Med), FCPS (Pulm), FCCP (USA). Associate Professor Pulmonology & Head Chest Unit-II, Ojha Institute of Chest Diseases Dow University of Health Sciences, Karachi, Pakistan; 3 Dr. Sagheer Hussain, MBBS. Chest Unit-II, Ojha Institute of Chest Diseases Dow University of Health Sciences, Karachi, Pakistan; 4 Dr. Syed Khalid Ali, MBBS, DTCD, MCPS (Pulm). Chest Unit-II, Ojha Institute of Chest Diseases Dow University of Health Sciences, Karachi, Pakistan

**Keywords:** Mortality, Smear positive, Pulmonary tuberculosis, Risk factors, Comorbidities

## Abstract

**Objective:**

To evaluate risk factors having significant effect on mortality of smear positive Pulmonary Tuberculosis (PTB) inpatients

**Methods:**

A descriptive cross-sectional study was conducted at Ojha Institute of Chest Diseases, Dow University Hospital Ojha Campus, Karachi. One hundred and seventy (170) inpatients of smear positive PTB confirmed by Acid Fast Bacilli (AFB) smear, aged between 13-80 years were enrolled by using consecutive sampling technique while patients with drug resistant Tuberculosis (TB) and extra pulmonary TB were excluded from the study. Selected patients were interviewed for collecting demographic data and risk factor data by using a standard questionnaire.

**Results:**

Out of 170 PTB inpatients, mortality was observed in 23 (13.5%) patients among which male patients were 12 (52.2%), and female were 11 (47.8%). Mortality was significantly associated with increasing age (p=0.003), socioeconomic status (p=0.019), anemia (p=0.03), Chronic Liver Disease (CLD) (p=0.005), Diabetes Mellitus (DM) (p=0.001), Human Immunodeficiency Virus (HIV) (p=0.007), Hypertension (HTN) (p=0.006), recurrent TB (p=0.001), and smoking (p=0.001).

**Conclusion:**

Increasing age, poverty, smoking history, and presence of comorbidities like DM, CLD, HIV, hypertension, and anemia are associated with higher mortality in smear positive PTB cases.

## INTRODUCTION

Tuberculosis (TB) is among the oldest infectious epidemic diseases, developed due to airborne bacterium i.e. Mycobacterium tuberculosis.^1^ It is multi-system infectious disease that mostly affect the lungs, commonly known as Pulmonary Tuberculosis (PTB). Tuberculosis affects the different body systems including respiratory, gastrointestinal, lymphoreticular, musculoskeletal, reproductive, and central nervous system.^2^ It is among the world’s major health related problems, affecting the one third population latently.^3^ World Health Organization (WHO) reports ten million new TB cases in 2017 including 5.8 million men, 3.2 million women, and one million children.^4^ Pakistan is ranked among leading eight countries in which 66.6% of new cases of PTB were reported.^4^ Human Immunodeficiency Virus (HIV) is a leading risk factor reported by WHO, that causes 11% of newly diagnosed PTB.^5^

Pulmonary Tuberculosis patients frequently present with cough with or without sputum, fever, weight loss, night sweats, breathlessness, and chest pain. Sputum smear test is the most commonly used diagnostic method for PTB patients.^6^ Smear positive PTB patients are considered as most infectious ones because these are capable of spreading infection to 15 non-PTB people per year. Smear test is a corner stone in early diagnosis of PTB in patients and to initiate the treatment with anti-tuberculosis therapy (ATT).^7^ Despite the fact, PTB is early diagnosed and treated but still the leading cause of mortality. WHO reports the 1.3 million and 0.3 million deaths throughout the world in 2017 due to HIV negative TB and HIV positive TB respectively.^4^ PTB mortality rate varies in different regions of world, developing countries with high mortality rate whereas developed countries with low mortality rate.^8^

However, PTB is directly associated with different risk factors that increases the mortality such as delay in diagnosis of PTB, poor compliance with ATT, lack of proper health facilities and ignorance of patients towards health in developing countries, homelessness, increasing age, HIV infection, anemia, DM, CLD,HTN, recurrent TB, and smoking. These several risk factors increase the chances of development of drug resistance that leads towards therapy failure, recurrence of PTB, and ultimately deaths of patients.^9^-^14^

The research objective of this study was evaluation of risk factors associated with mortality of smear positive PTB patients treated at Ojha institute of chest diseases.

## METHODS

A descriptive cross-sectional study was conducted at Ojha institute of Chest Disease, Dow University Hospital Ojha Campus Karachi. One hundred and seventy (170) inpatients of smear positive PTB patients, receiving ATT category I were selected in first four months of study and each patient was followed during intensive phase of treatment i.e., two months. Study period was from 1^st^ October 2018 to 31^st^ March 2019. PTB patients having age between 13-80 years were enrolled by using consecutive sampling technique and patients with drug resistant TB (on Gene Xpert) and extrapulmonary TB were excluded from the study. Research approval was obtained from Institutional Review Board (IRB) of Dow University of Health Sciences, Karachi. Selected patients were interviewed for collecting demographic data and associated risk factors like; anemia, CLD, DM, HIV, HTN, and history of smoking and PTB by using a standard questionnaire.

Socioeconomic status of patients was categorized into different classes on the basis of per month income in Pakistani Rupees (PKR), such as lower class (< 20000 PKR), middle class (20000-50000 PKR), and upper class (> 50000 PKR). Different risk factors were confirmed by using standard protocol, such as blood pressure of each PTB patient was measured for confirmation of hypertension. Specific laboratory tests were performed for confirming associated risk factors such as Complete Blood Count (CBC) for anemia, Fasting and Random blood sugar (RBS) for diabetes mellitus, HIV screening, ultrasound abdomen and Liver Function Test (LFT) for chronic liver disease. All collected data was interpreted with SPSS version 22, by applying chi-square test and using < 0.05 as significant p-value.

## RESULTS

During study period of six months, 170 patients of PTB were evaluated for different risk factors, among which majority of the patients were male 92 (54.1%), and 78 (45.9%) were female. Patients of all age groups were suffering from PTB, majority 58 (34.1%) in 13-30 years, followed by 43 (25.3%) in 46-60 years, 36 (21.2%) in 31-45 years, and 33 (19.4%) in 61-80 years. Majority of the patients belonged to lower socio economic class 95 (55.9%), followed by middle class 67 (39.4%), and only 8 (4.7%) from upper class. Most of the patients were illiterate 60 (35.3%), and only 14 (8.2%) were graduate. Most of the PTB patients were married 125 (73.5%), followed by 38 (22.4%) single (Table-I).

**Table I:** Demographic Data.

Variable	Frequency (n=170)	Percentage
***Gender***		
Male	92	54.1
Female	78	45.9
***Age (Years)***		
13-30	58	34.1
31-45	36	21.2
46-60	43	25.3
61-80	33	19.4
***Socioeconomic Status***		
Lower	95	55.9
Middle	67	39.4
Upper	8	4.7
***Marital Status***		
Single	38	22.4
Married	125	73.5
Divorced	2	1.2
Widowed	5	2.9
***Education***		
Illiterate	60	35.3
Primary	22	12.9
Middle	17	10.0
Matriculation	25	14.7
Intermediate	32	18.8
Graduate	14	8.2

At the time of enrollment in study, mostly patients presented with cough > 2 weeks 109 (64.1%), followed by weight loss 73 (42.9%), breathlessness 31 (18.2%), fever 27 (15.9%), chest pain 21 (12.4%), and night sweats in 13 (7.6%). Chest X-ray was normal in only 2.9%, and abnormal in 97.1%. Infiltrates in CXR was mostly observed in 55 (32.4%) patients followed by consolidation in 51 (30.4%), and cavitation in 39 (22.9%). PTB was unilateral in 101 (59.4%), and bilateral in 69 (40.6%) patients (Table-II). Most of the PTB inpatients i.e. 147 (86.5%) were managed and discharged (DC) from the hospital, whereas 23 (13.5%) PTB inpatients were expired during the treatment.

**Table II:** Clinical Presentation of PTB patients

Variable	Frequency (n=170)	Percentage
***Sign and symptoms***		
Cough	109	64.1
Fever	27	15.9
Weight Loss	73	42.9
Night sweats	13	7.6
Breathlessness	31	18.2
Chest pain	21	12.4
***CXR***		
Normal	5	2.9
Consolidation	51	30.0
Infiltrates	55	32.4
Cavitation	39	22.9
Consolidation + Infiltrate	3	1.8
Cavitation + Infiltrate	13	7.6
Consolidation + Cavitation	4	2.4
***Extensive Disease***		
Unilateral	101	59.4
Bilateral	69	40.6

After management of PTB inpatients, mortality in 23 PTB patients was observed and assessed for different risk factors, out of which 12 (52.2%), and 11 (47.8%) patients were male and female respectively. During study different risk factors were evaluated that shows significant relation with mortality such as increasing age (p=0.003), socioeconomic status (p=0.019), anemia (p=0.03), CLD (p=0.005), DM (p=0.001), HIV (p=0.007), HTN (p=0.006), recurrent TB (p=0.001), and smoking (p=0.001) (Table-III).

**Table III:** Risk factors of Mortality.

Variables	Outcome		Total (n=170)	P-value

	Treated & DC (n=147)	Expired (n=23)		
***Gender***				
Male	80 (54.4%)	12 (52.2%)	92 (54.1%)	0.8
Female	67 (45.6%)	11 (47.8%)	78 (45.9%)
***Age***				
13-30	55 (37.5%)	3 (13.0%)	58 (34.1%)	0.003
31-45	32 (21.8%)	4 (17.4%)	36 (21.2%)	
46-60	38 (25.8%)	5 (21.7%)	43 (25.3%)	
61-80	22 (14.9%)	11 (47.8%)	33 (19.4%)
***Socioeconomic status***				
Lower	76 (51.7%)	19 (82.6%)	95 (55.9%)	0.019
Middle	63 (62.9%)	4 (17.4%)	67 (39.4%)	
Upper	8 (5.4%)	0 (0.0%)	8 (4.7%)
***Educational status***				
Illiterate	49 (33.3%)	11 (47.8%)	60 (35.3%)	0.8
Primary	20 (13.6%)	2 (8.7%)	22 (12.9%)	
Middle	15 (10.2%)	2 (8.7%)	17 (10.0%)	
Matriculation	22 (15.0%)	3 (13.0%)	25 (14.7%)	
Intermediate	28 (19.0%)	4 (17.4%)	32 (18.8%)	
Graduate	13 (8.8%)	1 (4.3%)	14 (8.2%)
***Anemia***				
Yes	74 (50.3%)	17 (73.9%)	91 (53.5%)	0.03
No	73 (49.7%)	6 (26.1%)	79 (46.5%)
***CLD***				
Yes	18 (12.2%)	8 (34.8%)	26 (15.3%)	0.005
No	129 (87.8%)	15 (65.2%)	144 (84.7%)
***DM***				
Yes	17 (11.6%)	15 (65.2%)	32 (18.8%)	0.001
No	130 (88.4%)	8 (34.8%)	138 (81.2%)
***HIV***				
Yes	1 (0.7%)	2 (8.7%)	3 (1.8%)	0.007
No	146 (99.3%)	21 (91.3%)	167 (98.2%)
***HTN***				
Yes	8 (5.4%)	5 (21.7%)	13 (7.6%)	0.006
No	139 (94.6%)	18 (78.3%)	157 (92.4%)
***Recurrent TB***				
Yes	53 (36.1%)	1 (4.3%)	54 (31.8%)	0.001
No	94 (63.9%)	22 (95.7%)	116 (68.2%)
***Smoker***				
Yes	35 (23.8%)	16 (69.6%)	51 (30.0%)	0.001
No	112 (76.2%)	7 (30.4%)	119 (70.0%)	

## DISCUSSION

Pulmonary tuberculosis (PTB) is among the major health related problems in developing or low-income countries. Despite extensive development in medical field, and improvements in health-related facilities PTB is still among the life-threatening diseases in poor countries.^15^ Direct involvement of different risk factors for this dilemma are the distinct reason of increasing infection susceptibility, and PTB progression.^15^,^16^ These factors also play vital role in prediction of mortality in PTB patients, such as increasing age, poor health care facilities due to poverty, low income, anemia, CLD, DM, HIV, HTN, recurrent TB, and smoking.^17^,^18^

WHO reports that morbidity and mortality of TB is continuously increasing throughout the world, and TB is leading cause of mortality among infectious diseases.^4^,^5^ Current study have two important findings first one is the increased mortality rate in PTB patients, and second one is the significant relation of different risk factors with PTB.

First important finding of current study was the higher rate of mortality in PTB patients i.e., 13.5%, whereas most of the other studies reported the lower mortality prevalence than current study such as Asgedom SW et al., 11.3%,^17^ Shahrezaei M et al., 1.6%,^18^ and Rodrigo T et al., 3.5%.^19^ Another study by Takarinda KC et al. reported the higher prevalence of mortality 22.0% in TB patients.^20^ In current study mortality rate was high due to several reasons such as illiteracy, poverty, and presence of comorbidities.

Second important finding is the direct and significant relation of different risk factors with mortality in PTB patients. Mortality shows significant relation with increasing age, poverty, anemia, CLD, DM, HIV, HTN, recurrent TB, and smoking. Similar to our result different studies show the significance of various risk factors and mortality.^18^,^21^,^22^ A Pakistani study by Khaliq A et al. on different social and environmental risk factors in PTB patients reported the significant relation of PTB mortality with male gender, poverty, smoking, DM, asthma, HIV, and reoccurrence of TB.^21^ Study by Shahrezaei M et al. reported the significant relation of mortality with increasing age, weight, and TB case. Other important reported factors were DM, and HIV.^18^ Another study by Alavi-Naini R et al. reports the anemia, smoking, DM, HIV, drug abuse, hepatitis, and recurrent TB as a predictor for PTB mortality.^22^

In low income countries, poverty is the most common cause of increasing malnutrition and diseases such as TB. Malnutrition directly affects the cell mediated immunity i.e., the principal defense against TB resulting in rapid progression of TB in malnourished as well as in immunocompromised patients such as in HIV.^23^ Increasing age is another important factor that increases the comorbidities and makes the situation worse for appropriate management of TB, ultimately mortality rate increases. PTB patients suffering from DM are at a higher risk of developing TB with increased progression and mortality. TB is most commonly known as disease of poverty because of its direct association with other risk factors that increases the emergence and progression of TB. Current study also reports the higher prevalence of illiteracy, lower socioeconomic status, and presence of comorbid conditions, which are not only directly, associated with progression of PTB but also increase the chances of failure of therapy resulting in death of patients.^23^-^25^

### Limitation of the study

The follow up of patients was for short period of time i.e., intensive phase, and severity of comorbid conditions was not evaluated. Poor health care facilities, delay in diagnosis, non-compliance with treatment, and emergence of resistance can be further studied as associated risk factor of mortality in smear positive PTB patients.

## CONCLUSION

The study concludes that rate of mortality was high in PTB inpatients treated at a tertiary care hospital of Ojha Institute of Chest Diseases. Risk factors; such as increasing age, poverty, anemia, CLD, DM, HIV, HTN, recurrent TB, and smoking are significantly associated with mortality in these patients.

### Authors’ Contribution

**SHa** wrote manuscript, did statistical analysis.

**FFZ** designed and conceived the study, did statistical analysis and final approval of manuscript.

**SHu & SKA** collected data, literature search and did initial draft write-up.
